# Tetra­aqua­{1-[(1*H*-1,2,3-benzotriazol-1-yl)meth­yl]-1*H*-1,2,4-triazole}sulfato­zinc(II) dihydrate

**DOI:** 10.1107/S160053681004331X

**Published:** 2010-10-30

**Authors:** Yan-Zhi Wang, Xiao-Kun Li, Huai-Xia Yang, Wan Zhou, Xiang-Ru Meng

**Affiliations:** a Pharmacy College, Henan University of Traditional Chinese Medicine, Zhengzhou 450008, People’s Republic of China; b Department of Chemistry, Zhengzhou University, Zhengzhou 450052, People’s Republic of China

## Abstract

In the title complex, [Zn(SO_4_)(C_9_H_8_N_6_)(H_2_O)_4_]·2H_2_O, the Zn^II^ ion is six-coordinated by one N atom from a 1-[(1*H*-1,2,3-benzotriazol-1-yl)meth­yl]-1*H*-1,2,4-triazole ligand and five O atoms from one monodentate sulfate anion and four water mol­ecules in a distorted octa­hedral geometry. The sulfate tetra­hedron is rotationally disordered over two positions in a 0.618 (19):0.382 (19) ratio. In the crystal, adjacent mol­ecules are linked through O—H⋯O and O—H⋯N hydrogen bonds involving the cation, the anion, and the coordinated and uncoordinated water mol­ecules into a three-dimensional network.

## Related literature

For background to complexes based on symmetrical *N*-hetero­cyclic ligands, see: Fan & Hanson (2005 ▶); Zhao *et al.* (2007 ▶). For background to complexes with Zn^II^, see: Lin *et al.* (2008 ▶); Liu *et al.* (2010 ▶).
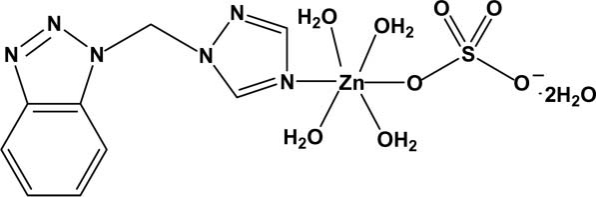


## Experimental

### 

#### Crystal data

[Zn(SO_4_)(C_9_H_8_N_6_)(H_2_O)_4_]·2H_2_O
*M* *_r_* = 469.74
Triclinic, 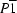

*a* = 7.5439 (15) Å
*b* = 7.9573 (16) Å
*c* = 16.151 (3) Å
α = 99.60 (3)°
β = 92.16 (3)°
γ = 112.24 (3)°
*V* = 879.4 (3) Å^3^
*Z* = 2
Mo *K*α radiation
μ = 1.58 mm^−1^
*T* = 293 K
0.24 × 0.23 × 0.21 mm

#### Data collection

Rigaku Saturn CCD diffractometer
Absorption correction: multi-scan (*CrystalClear*; Rigaku/MSC, 2006 ▶) *T*_min_ = 0.703, *T*_max_ = 0.733
7688 measured reflections
3442 independent reflections
3130 reflections with *I* > 2σ(*I*)
*R*_int_ = 0.018

#### Refinement

*R*[*F* ^2^ > 2σ(*F* ^2^)] = 0.028
*wR*(*F* ^2^) = 0.070
*S* = 1.04
3442 reflections
272 parameters
H-atom parameters constrained
Δρ_max_ = 0.29 e Å^−3^
Δρ_min_ = −0.27 e Å^−3^

### 

Data collection: *CrystalClear* (Rigaku/MSC, 2006 ▶); cell refinement: *CrystalClear*; data reduction: *CrystalClear*; program(s) used to solve structure: *SHELXL97* (Sheldrick, 2008 ▶); program(s) used to refine structure: *SHELXL97* (Sheldrick, 2008 ▶); molecular graphics: *SHELXTL* (Sheldrick, 2008 ▶); software used to prepare material for publication: *SHELXTL*.

## Supplementary Material

Crystal structure: contains datablocks global, I. DOI: 10.1107/S160053681004331X/wm2415sup1.cif

Structure factors: contains datablocks I. DOI: 10.1107/S160053681004331X/wm2415Isup2.hkl

Additional supplementary materials:  crystallographic information; 3D view; checkCIF report

## Figures and Tables

**Table 1:** Hydrogen-bond geometry (Å, °)

*D*—H⋯*A*	*D*—H	H⋯*A*	*D*⋯*A*	*D*—H⋯*A*
O8—H8*A*⋯O3′	0.85	2.29	2.793 (14)	118
O10—H10*A*⋯O1	0.85	2.09	2.938 (2)	178
O10—H10*A*⋯O2′	0.85	2.51	3.028 (8)	120
O5—H5*B*⋯O4^i^	0.85	1.94	2.761 (5)	163
O5—H5*B*⋯O4′^i^	0.85	2.19	2.988 (13)	156
O7—H7*B*⋯O1^i^	0.85	1.98	2.823 (2)	170
O5—H5*A*⋯O10^ii^	0.85	1.90	2.731 (2)	165
O6—H6*A*⋯O4^iii^	0.85	1.94	2.752 (5)	159
O6—H6*A*⋯O4′^iii^	0.85	1.94	2.778 (8)	171
O6—H6*B*⋯O10^iv^	0.85	1.96	2.808 (2)	172
O7—H7*A*⋯O2′^iv^	0.85	1.84	2.684 (7)	171
O7—H7*A*⋯O2^iv^	0.85	1.87	2.701 (4)	164
O8—H8*B*⋯O9^v^	0.85	1.82	2.673 (3)	177
O8—H8*A*⋯N2^vi^	0.85	2.37	3.122 (3)	148
O9—H9*B*⋯O3^vii^	0.85	2.03	2.837 (8)	159
O9—H9*B*⋯O2′^vii^	0.85	2.22	2.919 (17)	139
O9—H9*B*⋯O3′^vii^	0.85	2.48	3.266 (17)	154
O9—H9*A*⋯N6^viii^	0.85	2.01	2.854 (3)	174
O10—H10*B*⋯O2^ix^	0.85	1.99	2.806 (10)	159
O10—H10*B*⋯O4′^ix^	0.85	2.08	2.836 (15)	147

Symmetry codes: (i) 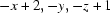; (ii) 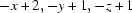; (iii) 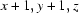; (iv) 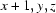; (v) 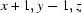; (vi) 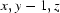; (vii) 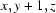; (viii) 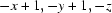; (ix) 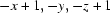.

**Fig. 1.**
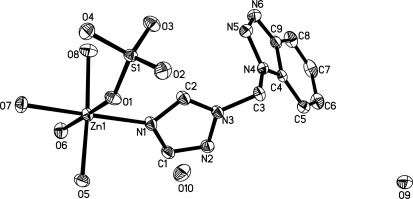
View of the title complex, showing the labelling of the atoms. Displacement ellipsoids are displayed at the 30% probability level. H atoms are omitted for clarity; only one orientation of the disordered SO4 tetrahedron is shown.

**Fig. 2.**
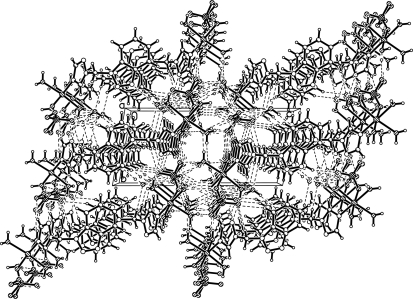
View of the title complex, showing the packing of the structure. Hydrogen bonds are indicated by dashed lines.

## supplementary crystallographic information

### Comment

Up to now, numerous complexes with one-, two- and three-dimensional structure motifs based on symmetrical N-heterocyclic ligands have been synthesized and reported (Fan & Hanson, 2005; Zhao *et al.*, 2007), whereas complexes based on unsymmetrical N-heterocyclic ligands are relatively scarce. Focused on complexes with Zn^II^, this ion is able to coordinate to different donors simultaneously and the final products can exhibit promising luminescent properties (Lin *et al.*, 2008; Liu *et al.*, 2010). In this work, through the reaction of 1-((benzotriazol-1-yl)methyl)-1-*H*-1,2,4-triazole (bmt) with zinc sulfate at room temperature, we obtained the title complex [Zn(bmt)(SO_4_)(H_2_O)_4_](H_2_O)_2_, which is reported here.

As shown in Figure 1, the Zn^II^ ion displays a distorted octahedral coordination defined by five oxygen atoms from four water molecules and one monodentate sulfate anion and by one nitrogen atom from the bmt ligand. Atoms O1, O5, O6, O8 and Zn1 are nearly co-planar (the mean deviation from the plane is 0.0258 Å), and atoms O7 and N1 are located in the apical positions. The SO_4_ tetrahedron is rotationally disordered about its S—O axis passing through O1 and S1 atoms. O—H···O and O—H···N hydrogen bonds including coordinated and uncoordinated water molecules, the cations and anions consolidate the crystal packing (Figure 2).

### Experimental

The ligand 1-((benzotriazol-1-yl)methyl)-1-*H*-1,2,4-triazole (0.1 mmol) in methanol (5 ml) was added dropwise to an aqueous solution (2 ml) of zinc sulfate (0.1 mmol). The resulting solution was allowed to stand at room temperature. After three weeks, colorless crystals with good quality were obtained from the filtrate and were dried in air.

### Refinement

The disordered sulfate anion has been modeled by splitting it into two combined parts (O2, O3, O4 and O2', O3', O4'), the site occupation factors of which refined in a ratio of 0.618 (19):0.382 (19). H atoms are positioned geometrically and refined as riding atoms, with C-H = 0.93 (aromatic) and 0.97 (CH_2_) Å and O-H = 0.85 Å, and with *U*_iso_(H) = 1.2 *U*_eq_(C,O).

### Crystal data

**Table:**

[Zn(SO_4_)(C_9_H_8_N_6_)(H_2_O)_4_]·2H_2_O	*Z* = 2
*M**_r_* = 469.74	*F*(000) = 484
Triclinic, *P*1	*D*_x_ = 1.774 Mg m^−^^3^
Hall symbol: -P 1	Mo *K*α radiation, λ = 0.71073 Å
*a* = 7.5439 (15) Å	Cell parameters from 2915 reflections
*b* = 7.9573 (16) Å	θ = 2.6–27.9°
*c* = 16.151 (3) Å	µ = 1.58 mm^−^^1^
α = 99.60 (3)°	*T* = 293 K
β = 92.16 (3)°	Prism, colourless
γ = 112.24 (3)°	0.24 × 0.23 × 0.21 mm
*V* = 879.4 (3) Å^3^	

### Data collection

**Table:**

Rigaku Saturn CCD diffractometer	3442 independent reflections
Radiation source: fine-focus sealed tube	3130 reflections with *I* > 2σ(*I*)
graphite	*R*_int_ = 0.018
Detector resolution: 28.5714 pixels mm^-1^	θ_max_ = 26.0°, θ_min_ = 2.6°
ω scans	*h* = −9→9
Absorption correction: multi-scan (*CrystalClear*; Rigaku/MSC, 2006)	*k* = −9→8
*T*_min_ = 0.703, *T*_max_ = 0.733	*l* = −19→19
7688 measured reflections	

### Refinement

**Table:**

Refinement on *F*^2^	Primary atom site location: structure-invariant direct methods
Least-squares matrix: full	Secondary atom site location: difference Fourier map
*R*[*F*^2^ > 2σ(*F*^2^)] = 0.028	Hydrogen site location: inferred from neighbouring sites
*wR*(*F*^2^) = 0.070	H-atom parameters constrained
*S* = 1.04	*w* = 1/[σ^2^(*F*_o_^2^) + (0.0343*P*)^2^ + 0.4616*P*] where *P* = (*F*_o_^2^ + 2*F*_c_^2^)/3
3442 reflections	(Δ/σ)_max_ = 0.001
272 parameters	Δρ_max_ = 0.29 e Å^−^^3^
0 restraints	Δρ_min_ = −0.27 e Å^−^^3^

### Special details

**Table:**

Geometry. All e.s.d.'s (except the e.s.d. in the dihedral angle between two l.s. planes) are estimated using the full covariance matrix. The cell e.s.d.'s are taken into account individually in the estimation of e.s.d.'s in distances, angles and torsion angles; correlations between e.s.d.'s in cell parameters are only used when they are defined by crystal symmetry. An approximate (isotropic) treatment of cell e.s.d.'s is used for estimating e.s.d.'s involving l.s. planes.
Refinement. Refinement of *F*^2^ against ALL reflections. The weighted *R*-factor *wR* and goodness of fit *S* are based on *F*^2^, conventional *R*-factors *R* are based on *F*, with *F* set to zero for negative *F*^2^. The threshold expression of *F*^2^ > σ(*F*^2^) is used only for calculating *R*-factors(gt) *etc*. and is not relevant to the choice of reflections for refinement. *R*-factors based on *F*^2^ are statistically about twice as large as those based on *F*, and *R*- factors based on ALL data will be even larger.

### Fractional atomic coordinates and isotropic or equivalent isotropic displacement parameters (Å^2^)

**Table:**

	*x*	*y*	*z*	*U*_iso_*/*U*_eq_	Occ. (<1)
Zn1	1.09002 (3)	0.17235 (3)	0.371640 (15)	0.02549 (9)	
S1	0.62271 (7)	−0.14764 (7)	0.35463 (3)	0.02418 (12)	
O1	0.7976 (2)	0.0142 (2)	0.39731 (9)	0.0305 (3)	
O2	0.4569 (4)	−0.1335 (9)	0.3924 (5)	0.0409 (19)	0.618 (19)
O3	0.6021 (13)	−0.1670 (12)	0.2661 (5)	0.0369 (14)	0.618 (19)
O4	0.6445 (10)	−0.3168 (6)	0.3758 (5)	0.0414 (14)	0.618 (19)
O2'	0.4634 (9)	−0.0755 (15)	0.3547 (9)	0.057 (3)	0.382 (19)
O3'	0.656 (2)	−0.189 (2)	0.2635 (9)	0.043 (3)	0.382 (19)
O4'	0.574 (2)	−0.2951 (11)	0.3977 (6)	0.044 (3)	0.382 (19)
O5	1.1162 (2)	0.3281 (2)	0.49352 (9)	0.0349 (4)	
H5B	1.1754	0.3012	0.5318	0.042*	
H5A	1.1677	0.4457	0.5028	0.042*	
O6	1.3836 (2)	0.3225 (2)	0.35396 (11)	0.0375 (4)	
H6A	1.4383	0.4397	0.3616	0.045*	
H6B	1.4681	0.3082	0.3852	0.045*	
O7	1.1822 (2)	−0.0011 (2)	0.42915 (10)	0.0342 (4)	
H7A	1.2706	−0.0324	0.4095	0.041*	
H7B	1.1743	−0.0138	0.4803	0.041*	
O8	1.0517 (2)	−0.0077 (2)	0.25848 (10)	0.0378 (4)	
H8B	1.1514	−0.0103	0.2365	0.045*	
H8A	0.9778	−0.1201	0.2564	0.045*	
N1	0.9899 (3)	0.3421 (2)	0.31394 (11)	0.0288 (4)	
N2	0.9472 (3)	0.5789 (2)	0.27075 (12)	0.0344 (4)	
N3	0.7996 (2)	0.4154 (2)	0.23720 (11)	0.0265 (4)	
N4	0.6884 (3)	0.4047 (2)	0.09507 (11)	0.0285 (4)	
N5	0.6883 (3)	0.2466 (3)	0.04821 (13)	0.0395 (5)	
N6	0.7300 (3)	0.2763 (3)	−0.02657 (13)	0.0419 (5)	
C1	1.0571 (3)	0.5274 (3)	0.31691 (14)	0.0328 (5)	
H1	1.1710	0.6106	0.3488	0.039*	
C2	0.8284 (3)	0.2775 (3)	0.26262 (14)	0.0324 (5)	
H2	0.7469	0.1533	0.2467	0.039*	
C3	0.6397 (3)	0.4063 (3)	0.18067 (13)	0.0308 (5)	
H3A	0.5276	0.2952	0.1823	0.037*	
H3B	0.6073	0.5123	0.1997	0.037*	
C4	0.7304 (3)	0.5407 (3)	0.04844 (13)	0.0272 (4)	
C5	0.7419 (3)	0.7219 (3)	0.06513 (15)	0.0349 (5)	
H5	0.7230	0.7772	0.1176	0.042*	
C6	0.7834 (4)	0.8139 (4)	−0.00128 (18)	0.0442 (6)	
H6	0.7918	0.9352	0.0066	0.053*	
C7	0.8134 (4)	0.7313 (4)	−0.08015 (18)	0.0495 (7)	
H7	0.8431	0.8000	−0.1227	0.059*	
C8	0.8003 (4)	0.5530 (4)	−0.09633 (16)	0.0452 (6)	
H8	0.8186	0.4981	−0.1490	0.054*	
C9	0.7578 (3)	0.4563 (3)	−0.02981 (14)	0.0334 (5)	
O9	0.3572 (3)	0.9693 (2)	0.18654 (11)	0.0443 (4)	
H9B	0.4072	0.9201	0.2182	0.053*	
H9A	0.3326	0.9027	0.1373	0.053*	
O10	0.6604 (2)	0.3016 (2)	0.46855 (11)	0.0408 (4)	
H10A	0.6979	0.2174	0.4471	0.049*	
H10B	0.6070	0.2662	0.5115	0.049*	

### Atomic displacement parameters (Å^2^)

**Table:**

	*U*^11^	*U*^22^	*U*^33^	*U*^12^	*U*^13^	*U*^23^
Zn1	0.02551 (14)	0.02764 (14)	0.02428 (14)	0.01074 (10)	0.00097 (9)	0.00719 (9)
S1	0.0209 (3)	0.0230 (3)	0.0249 (3)	0.0055 (2)	0.00115 (19)	0.00244 (19)
O1	0.0240 (8)	0.0301 (8)	0.0281 (8)	0.0012 (6)	0.0017 (6)	0.0035 (6)
O2	0.0209 (14)	0.041 (3)	0.056 (3)	0.0108 (14)	0.0063 (15)	0.0004 (19)
O3	0.037 (4)	0.047 (2)	0.024 (2)	0.014 (2)	0.002 (2)	0.0040 (16)
O4	0.047 (3)	0.0268 (17)	0.050 (3)	0.0147 (17)	−0.006 (2)	0.0086 (16)
O2'	0.038 (3)	0.072 (4)	0.081 (6)	0.038 (3)	0.017 (3)	0.030 (5)
O3'	0.033 (6)	0.056 (5)	0.028 (3)	0.010 (4)	0.008 (4)	−0.010 (3)
O4'	0.059 (6)	0.025 (3)	0.040 (4)	0.005 (3)	0.002 (3)	0.012 (2)
O5	0.0417 (9)	0.0287 (8)	0.0281 (8)	0.0092 (7)	−0.0044 (7)	0.0019 (6)
O6	0.0274 (8)	0.0326 (9)	0.0499 (10)	0.0066 (7)	0.0016 (7)	0.0138 (7)
O7	0.0405 (9)	0.0412 (9)	0.0315 (8)	0.0248 (8)	0.0071 (7)	0.0136 (7)
O8	0.0347 (9)	0.0374 (9)	0.0307 (9)	0.0050 (7)	0.0065 (7)	0.0000 (7)
N1	0.0273 (9)	0.0283 (9)	0.0295 (10)	0.0079 (8)	−0.0017 (8)	0.0102 (7)
N2	0.0347 (10)	0.0249 (9)	0.0392 (11)	0.0083 (8)	−0.0037 (8)	0.0046 (8)
N3	0.0269 (9)	0.0266 (9)	0.0244 (9)	0.0079 (8)	−0.0015 (7)	0.0075 (7)
N4	0.0327 (10)	0.0301 (10)	0.0254 (9)	0.0161 (8)	−0.0012 (7)	0.0047 (7)
N5	0.0481 (12)	0.0356 (11)	0.0374 (12)	0.0224 (10)	−0.0033 (9)	0.0012 (9)
N6	0.0474 (13)	0.0468 (12)	0.0338 (11)	0.0259 (10)	−0.0003 (9)	−0.0029 (9)
C1	0.0302 (12)	0.0288 (11)	0.0337 (12)	0.0072 (10)	−0.0057 (9)	0.0035 (9)
C2	0.0307 (12)	0.0258 (11)	0.0349 (12)	0.0038 (9)	−0.0045 (9)	0.0096 (9)
C3	0.0276 (11)	0.0376 (12)	0.0284 (11)	0.0131 (10)	−0.0007 (9)	0.0100 (9)
C4	0.0228 (10)	0.0333 (11)	0.0253 (11)	0.0109 (9)	−0.0022 (8)	0.0064 (9)
C5	0.0342 (13)	0.0329 (12)	0.0358 (13)	0.0125 (10)	−0.0006 (10)	0.0041 (9)
C6	0.0396 (14)	0.0378 (14)	0.0557 (17)	0.0112 (11)	0.0003 (12)	0.0208 (12)
C7	0.0378 (14)	0.0657 (19)	0.0470 (16)	0.0131 (13)	0.0049 (12)	0.0333 (14)
C8	0.0404 (14)	0.0727 (19)	0.0276 (12)	0.0246 (14)	0.0088 (10)	0.0158 (12)
C9	0.0295 (11)	0.0442 (13)	0.0269 (11)	0.0169 (10)	−0.0010 (9)	0.0029 (9)
O9	0.0512 (11)	0.0463 (10)	0.0343 (9)	0.0215 (9)	0.0003 (8)	0.0003 (7)
O10	0.0422 (10)	0.0314 (9)	0.0502 (10)	0.0153 (8)	0.0090 (8)	0.0087 (7)

### Geometric parameters (Å, °)

**Table:**

Zn1—O8	2.0615 (17)	N3—C2	1.320 (3)
Zn1—O7	2.0869 (15)	N3—C3	1.457 (3)
Zn1—N1	2.0979 (18)	N4—N5	1.356 (3)
Zn1—O5	2.1028 (17)	N4—C4	1.367 (3)
Zn1—O6	2.1385 (18)	N4—C3	1.443 (3)
Zn1—O1	2.1824 (16)	N5—N6	1.297 (3)
S1—O4'	1.401 (7)	N6—C9	1.377 (3)
S1—O3	1.409 (8)	C1—H1	0.9300
S1—O2	1.446 (3)	C2—H2	0.9300
S1—O1	1.4912 (16)	C3—H3A	0.9700
S1—O3'	1.505 (13)	C3—H3B	0.9700
S1—O4	1.507 (4)	C4—C5	1.390 (3)
S1—O2'	1.516 (7)	C4—C9	1.390 (3)
O5—H5B	0.8500	C5—C6	1.378 (3)
O5—H5A	0.8500	C5—H5	0.9300
O6—H6A	0.8499	C6—C7	1.400 (4)
O6—H6B	0.8499	C6—H6	0.9300
O7—H7A	0.8500	C7—C8	1.364 (4)
O7—H7B	0.8501	C7—H7	0.9300
O8—H8B	0.8500	C8—C9	1.401 (3)
O8—H8A	0.8500	C8—H8	0.9300
N1—C2	1.321 (3)	O9—H9B	0.8499
N1—C1	1.357 (3)	O9—H9A	0.8501
N2—C1	1.313 (3)	O10—H10A	0.8499
N2—N3	1.360 (3)	O10—H10B	0.8500
			
O8—Zn1—O7	87.82 (7)	Zn1—O8—H8A	116.6
O8—Zn1—N1	91.97 (7)	H8B—O8—H8A	105.9
O7—Zn1—N1	178.51 (7)	C2—N1—C1	103.23 (18)
O8—Zn1—O5	173.22 (6)	C2—N1—Zn1	123.15 (15)
O7—Zn1—O5	86.93 (6)	C1—N1—Zn1	133.61 (15)
N1—Zn1—O5	93.18 (7)	C1—N2—N3	102.46 (17)
O8—Zn1—O6	90.69 (7)	C2—N3—N2	110.19 (17)
O7—Zn1—O6	88.10 (7)	C2—N3—C3	128.18 (18)
N1—Zn1—O6	93.38 (7)	N2—N3—C3	121.62 (17)
O5—Zn1—O6	93.40 (8)	N5—N4—C4	110.63 (18)
O8—Zn1—O1	91.05 (7)	N5—N4—C3	119.25 (18)
O7—Zn1—O1	88.44 (7)	C4—N4—C3	130.06 (18)
N1—Zn1—O1	90.10 (7)	N6—N5—N4	108.29 (19)
O5—Zn1—O1	84.54 (7)	N5—N6—C9	108.93 (19)
O6—Zn1—O1	176.06 (6)	N2—C1—N1	114.12 (19)
O4'—S1—O3	124.8 (5)	N2—C1—H1	122.9
O4'—S1—O2	79.7 (5)	N1—C1—H1	122.9
O3—S1—O2	112.6 (3)	N3—C2—N1	109.99 (19)
O4'—S1—O1	112.3 (3)	N3—C2—H2	125.0
O3—S1—O1	113.5 (4)	N1—C2—H2	125.0
O2—S1—O1	108.19 (15)	N4—C3—N3	111.05 (18)
O4'—S1—O3'	116.6 (7)	N4—C3—H3A	109.4
O3—S1—O3'	19.3 (5)	N3—C3—H3A	109.4
O2—S1—O3'	131.0 (4)	N4—C3—H3B	109.4
O1—S1—O3'	106.7 (6)	N3—C3—H3B	109.4
O4'—S1—O4	27.9 (4)	H3A—C3—H3B	108.0
O3—S1—O4	108.6 (4)	N4—C4—C5	133.5 (2)
O2—S1—O4	107.4 (2)	N4—C4—C9	103.73 (19)
O1—S1—O4	106.24 (19)	C5—C4—C9	122.8 (2)
O3'—S1—O4	94.5 (6)	C6—C5—C4	115.5 (2)
O4'—S1—O2'	109.8 (4)	C6—C5—H5	122.3
O3—S1—O2'	86.4 (4)	C4—C5—H5	122.3
O2—S1—O2'	31.9 (3)	C5—C6—C7	122.4 (2)
O1—S1—O2'	104.9 (3)	C5—C6—H6	118.8
O3'—S1—O2'	105.8 (5)	C7—C6—H6	118.8
O4—S1—O2'	135.7 (4)	C8—C7—C6	121.8 (2)
S1—O1—Zn1	138.82 (9)	C8—C7—H7	119.1
Zn1—O5—H5B	114.8	C6—C7—H7	119.1
Zn1—O5—H5A	120.7	C7—C8—C9	116.9 (2)
H5B—O5—H5A	103.1	C7—C8—H8	121.6
Zn1—O6—H6A	124.8	C9—C8—H8	121.6
Zn1—O6—H6B	116.0	N6—C9—C4	108.4 (2)
H6A—O6—H6B	96.0	N6—C9—C8	130.9 (2)
Zn1—O7—H7A	119.6	C4—C9—C8	120.7 (2)
Zn1—O7—H7B	126.2	H9B—O9—H9A	107.2
H7A—O7—H7B	110.1	H10A—O10—H10B	105.2
Zn1—O8—H8B	118.1		
			
O4'—S1—O1—Zn1	116.9 (8)	C2—N1—C1—N2	−0.1 (3)
O3—S1—O1—Zn1	−31.3 (4)	Zn1—N1—C1—N2	−179.33 (16)
O2—S1—O1—Zn1	−157.0 (4)	N2—N3—C2—N1	1.1 (3)
O3'—S1—O1—Zn1	−12.0 (7)	C3—N3—C2—N1	−179.8 (2)
O4—S1—O1—Zn1	88.0 (4)	C1—N1—C2—N3	−0.6 (3)
O2'—S1—O1—Zn1	−123.9 (7)	Zn1—N1—C2—N3	178.71 (14)
O8—Zn1—O1—S1	−4.72 (15)	N5—N4—C3—N3	76.6 (2)
O7—Zn1—O1—S1	−92.51 (15)	C4—N4—C3—N3	−106.4 (2)
N1—Zn1—O1—S1	87.25 (15)	C2—N3—C3—N4	−95.9 (3)
O5—Zn1—O1—S1	−179.57 (15)	N2—N3—C3—N4	83.0 (2)
O6—Zn1—O1—S1	−120.9 (8)	N5—N4—C4—C5	177.5 (2)
O8—Zn1—N1—C2	49.15 (19)	C3—N4—C4—C5	0.3 (4)
O7—Zn1—N1—C2	−33 (3)	N5—N4—C4—C9	−0.6 (2)
O5—Zn1—N1—C2	−126.43 (19)	C3—N4—C4—C9	−177.8 (2)
O6—Zn1—N1—C2	139.96 (19)	N4—C4—C5—C6	−177.9 (2)
O1—Zn1—N1—C2	−41.90 (19)	C9—C4—C5—C6	−0.2 (3)
O8—Zn1—N1—C1	−131.7 (2)	C4—C5—C6—C7	−0.5 (4)
O7—Zn1—N1—C1	147 (2)	C5—C6—C7—C8	1.0 (4)
O5—Zn1—N1—C1	52.7 (2)	C6—C7—C8—C9	−0.9 (4)
O6—Zn1—N1—C1	−40.9 (2)	N5—N6—C9—C4	−0.2 (3)
O1—Zn1—N1—C1	137.2 (2)	N5—N6—C9—C8	−178.1 (2)
C1—N2—N3—C2	−1.1 (2)	N4—C4—C9—N6	0.4 (2)
C1—N2—N3—C3	179.8 (2)	C5—C4—C9—N6	−177.9 (2)
C4—N4—N5—N6	0.5 (2)	N4—C4—C9—C8	178.7 (2)
C3—N4—N5—N6	178.03 (19)	C5—C4—C9—C8	0.4 (3)
N4—N5—N6—C9	−0.2 (3)	C7—C8—C9—N6	177.9 (2)
N3—N2—C1—N1	0.7 (3)	C7—C8—C9—C4	0.2 (4)

### Hydrogen-bond geometry (Å, °)

**Table:**

*D*—H···*A*	*D*—H	H···*A*	*D*···*A*	*D*—H···*A*
O8—H8A···O3'	0.85	2.29	2.793 (14)	118
O10—H10A···O1	0.85	2.09	2.938 (2)	178
O10—H10A···O2'	0.85	2.51	3.028 (8)	120
O5—H5B···O4^i^	0.85	1.94	2.761 (5)	163
O5—H5B···O4'^i^	0.85	2.19	2.988 (13)	156
O7—H7B···O1^i^	0.85	1.98	2.823 (2)	170
O5—H5A···O10^ii^	0.85	1.90	2.731 (2)	165
O6—H6A···O4^iii^	0.85	1.94	2.752 (5)	159
O6—H6A···O4'^iii^	0.85	1.94	2.778 (8)	171
O6—H6B···O10^iv^	0.85	1.96	2.808 (2)	172
O7—H7A···O2'^iv^	0.85	1.84	2.684 (7)	171
O7—H7A···O2^iv^	0.85	1.87	2.701 (4)	164
O8—H8B···O9^v^	0.85	1.82	2.673 (3)	177
O8—H8A···N2^vi^	0.85	2.37	3.122 (3)	148
O9—H9B···O3^vii^	0.85	2.03	2.837 (8)	159
O9—H9B···O2'^vii^	0.85	2.22	2.919 (17)	139
O9—H9B···O3'^vii^	0.85	2.48	3.266 (17)	154
O9—H9A···N6^viii^	0.85	2.01	2.854 (3)	174
O10—H10B···O2^ix^	0.85	1.99	2.806 (10)	159
O10—H10B···O4'^ix^	0.85	2.08	2.836 (15)	147

Symmetry codes: (i) −*x*+2, −*y*, −*z*+1; (ii) −*x*+2, −*y*+1, −*z*+1; (iii) *x*+1, *y*+1, *z*; (iv) *x*+1, *y*, *z*; (v) *x*+1, *y*−1, *z*; (vi) *x*, *y*−1, *z*; (vii) *x*, *y*+1, *z*; (viii) −*x*+1, −*y*+1, −*z*; (ix) −*x*+1, −*y*, −*z*+1.
